# Transactions of the Medical Association of Southern Central New York, at the 12th, 13th, 14th, and 15th Annual Meetings, Held in 1858 ’59 ’60, and 61

**Published:** 1863

**Authors:** 


Transactions of the Medical Association of Southern Central New York, at the 12th, 13th, 14th, and 15th Annual Meetings, held in 1858, '59, '60, and '61. Binghamton: Wm. S. Lawyer, Printer, 1863.

This is a pamphlet of 80 pages, neatly printed on fair paper. It contains the record of proceedings of four Annual Meetings of the Society, and several of the more interesting and directly practical papers read at the several meetings. We regret that the funds in the treasury did not permit the publication of all the papers and addresses which have been read to the Society. What has been selected, however, is of practical value, and will be read with interest.

